# Differential expression of genes mapping to recurrently abnormal chromosomal regions characterize neuroblastic tumours with distinct ploidy status

**DOI:** 10.1186/1755-8794-1-36

**Published:** 2008-08-13

**Authors:** Cinzia Lavarino, Idoia Garcia, Carlos Mackintosh, Nai-Kong V Cheung, Gema Domenech, José Ríos, Noelia Perez, Eva Rodríguez, Carmen de Torres, William L Gerald, Esperanza Tuset, Sandra Acosta, Helena Beleta, Enrique de Álava, Jaume Mora

**Affiliations:** 1 Developmental Tumour Biology Laboratory, Hospital Sant Joan de Déu, Fundació Sant Joan de Déu, Barcelona, Spain; 2 Pathology, Hospital Sant Joan de Déu, Fundació Sant Joan de Déu, Barcelona, Spain; 3 Hematology, Hospital Sant Joan de Déu, Fundació Sant Joan de Déu, Barcelona, Spain; 4 Molecular Pathology Laboratory, Centro de Investigación del Cáncer-IBMCC (USAL-CSIC), Salamanca, Spain; 5 Department of Pediatrics, Memorial Sloan-Kettering Cancer Centre, New York, USA; 6 Pathology, Memorial Sloan-Kettering Cancer Centre, New York, USA; 7 Unit of Biostatistics and Epidemiology, Universitat Autònoma, Barcelona, Spain

## Abstract

**Background:**

Neuroblastic tumours (NBTs) represent a heterogeneous spectrum of neoplastic diseases associated with multiple genetic alterations. Structural and numerical chromosomal changes are frequent and are predictive parameters of NBTs outcome. We performed a comparative analysis of the biological entities constituted by NBTs with different ploidy status.

**Methods:**

Gene expression profiling of 49 diagnostic primary NBTs with ploidy data was performed using oligonucleotide microarray. Further analyses using Quantitative Real-Time Polymerase Chain Reaction (Q-PCR); array-Comparative Genomic Hybridization (aCGH); and Fluorescent *in situ *Hybridization (FISH) were performed to investigate the correlation between aneuploidy, chromosomal changes and gene expression profiles.

**Results:**

Gene expression profiling of 49 primary near-triploid and near-diploid/tetraploid NBTs revealed distinct expression profiles associated with each NBT subgroup. A statistically significant portion of genes mapped to 1p36 (*P *= 0.01) and 17p13-q21 (*P *< 0.0001), described as recurrently altered in NBTs. Over 90% of these genes showed higher expression in near-triploid NBTs and the majority are involved in cell differentiation pathways. Specific chromosomal abnormalities observed in NBTs, 1p loss, 17q and whole chromosome 17 gains, were reflected in the gene expression profiles. Comparison between gene copy number and expression levels suggests that differential expression might be only partly dependent on gene copy number. Intratumoural clonal heterogeneity was observed in all NBTs, with marked interclonal variability in near-diploid/tetraploid tumours.

**Conclusion:**

NBTs with different cellular DNA content display distinct transcriptional profiles with a significant portion of differentially expressed genes mapping to specific chromosomal regions known to be associated with outcome. Furthermore, our results demonstrate that these specific genetic abnormalities are highly heterogeneous in all NBTs, and suggest that NBTs with different ploidy status may result from different mechanisms of aneuploidy driving tumourigenesis.

## Background

Neuroblastic tumours (NBTs) are one of the most common neoplasms in childhood, accounting for approximately 40% of solid tumours encountered in the first four years of life [1]. NBTs are heterogeneous in terms of their biological, genetic and morphological characteristics and exhibit marked diverse clinical behaviours.

The biological bases of these processes are poorly understood. There is an apparent link between NBTs aggressiveness and specific genetic aberrations (i.e., MYCN amplification, chromosome deletions of 1p36, 11q23, 14q32 or 19q13.3; gain of 17q and near-diploid/tetraploid DNA content), indicating that specific genetic alterations are present in individual categories of NBTs and likely contribute to clinical outcome [2-4].

Abnormal cellular DNA content is ubiquitous in cancer and has been linked to the rate of cell proliferation, cell differentiation, and prognosis in a variety of tumour cell types. In contrast to most other tumours, hyperploidy confers a favourable prognosis in NBTs [5], acute lymphoblastic leukemia [6], and rhabdomyosarcoma [7]. Non-metastatic loco-regional NBTs (stages 1, 2 and 3) often show modal chromosomal numbers in the near-triploid range (58 to 80 modal chromosome number) and few structural aberrations [5]. On the other hand, karyotypes of metastatic NBTs are commonly near-diploid (44 to 57 chromosomes) or near-tetraploid (81–103 chromosomes) with structural changes [5].

The presence of specific and recurrent chromosomal alterations in NBTs suggests that gene copy number abnormalities represent a major biologically relevant event, which contributes to NBT growth and survival. The aim of the current study was to gain further insight into the difference in gene expression of distinct biological entities within NBTs defined by the ploidy status.

## Methods

### Patients and samples

Forty-nine diagnostic primary NBT specimens (24 stages 1, 2, and 3; 7 stage 4s; and 18 stage 4) obtained from patients diagnosed and treated at MSKCC were selected for gene expression profiling (Table 1). Risk assessment was defined by the INSS staging classification, the MSKCC biological risk stratification criteria, and the COG clinical staging criteria. NBT stages 1, 2, 3 and 4s were treated without use of cytotoxic therapy, when possible, according to MSKCC protocols. Stage 4 NBTs patients were treated according to N5, N6 or N7 protocols. This study was approved by the MSKCC and HSJD Institutional Review Boards and informed consent was obtained before collection of all samples.

**Table 1:** Clinical and Biological characteristics of patients with Neuroblastoma evaluated according to tumour ploidy status.

Case number	ploidy	Age	INSS stage	MYCN amplification	Disease Status	Survival Status	microarray analysis	validation analysis
		<12m=0; >12m=1	1,2,3,4s=0; 4=1					

1	near-3n	1	0	NA	NP	A	Y	Y
2	near-3n	1	0	NA	NP	A	Y	.
3	near-3n	1	0	NA	NP	A	Y	Y
4	near-3n	0	0	NA	NP	A	Y	.
5	near-3n	0	0	NA	NP	A	Y	Y
6	near-3n	0	0	NA	NP	A	Y	.
7	near-3n	0	0	NA	P	A	Y	.
8	near-3n	0	0	NA	P	A	Y	.
9	near-3n	0	0	NA	NP	A	Y	.
10	near-3n	0	0	NA	NP	A	Y	.
11	near-3n	1	0	NA	NP	A	Y	Y
12	near-3n	0	0	NA	NP	A	Y	.
13	near-3n	1	1	NA	P	D	Y	.
14	near-3n	1	0	NA	NP	A	Y	.
15	near-3n	0	0	NA	NP	A	Y	Y
16	near-3n	0	0	NA	NP	A	Y	.
17	near-3n	0	0	NA	P	A	Y	Y
18	near-3n	1	1	NA	P	D	Y	Y
19	near-3n	1	0	NA	NP	A	Y	.
20	near-3n	1	0	NA	NP	A	Y	Y
21	near-3n	0	0	NA	P	A	Y	.
22	near-3n	1	0	NA	P	D	Y	Y
23	near-3n	0	0	NA	NP	A	.	Y
24	near-3n	0	0	NA	NP	A	.	Y
25	near-3n	0	0	NA	NP	A	.	Y
26	near-3n	0	0	NA	NP	A	.	Y
27	near-3n	1	0	NA	NP	A	.	Y
28	near-3n	1	0	NA	NP	A	.	Y
29	near-3n	1	0	NA	NP	A	.	Y
30	near-3n	1	0	NA	NP	A	.	Y
31	near-3n	0	1	NA	NP	A	.	Y
32	near-3n	0	0	NA	NP	A	.	Y
33	near-3n	1	1	NA	P	A	.	Y
34	near-3n	0	0	NA	NP	A	.	Y
35	near-3n	0	0	NA	P	D	.	Y
36	near-3n	0	0	NA	NP	A	.	Y
37	near-3n	0	0	NA	NP	A	.	Y

38	near-2n	1	1	A	NP	A	Y	Y
39	near-2n	1	1	A	P	D	Y	Y
40	near-2n	1	0	NA	P	D	Y	.
41	near-2n	1	1	A	P	D	Y	Y
42	near-2n	1	1	A	P	A	Y	Y
43	near-2n	1	1	NA	NP	A	Y	Y
44	near-2n	1	1	NA	P	D	Y	.
45	near-2n	1	1	A	P	D	Y	.
46	near-2n	1	1	NA	NP	A	Y	.
47	near-2n	1	0	NA	P	D	Y	.
48	near-2n	1	0	NA	P	D	Y	.
49	near-2n	0	1	A	NP	A	Y	.
50	near-2n	0	0	NA	NP	A	Y	Y
51	near-2n	0	0	NA	P	A	Y	.
52	near-2n	1	1	A	P	D	Y	Y
53	near-2n	1	0	A	NP	A	Y	Y
54	near-2n	1	0	NA	NP	A	Y	.
55	near-2n	1	1	NA	P	D	Y	Y
56	near-2n	0	0	NA	P	A	Y	.
57	near-2n	0	0	NA	NP	A	Y	.
58	near-2n	1	1	NA	P	D	Y	.
59	near-2n	1	1	NA	P	D	Y	Y
60	near-2n	1	0	NA	P	D	Y	.
61	near-2n	0	0	NA	NP	A	.	Y
62	near-2n	1	1	NA	P	D	.	Y
63	near-2n	1	1	A	P	D	.	Y
64	near-2n	1	1	NA	NP	A	.	Y
65	near-2n	1	1	NA	P	D	.	Y
66	near-2n	0	0	A	NP	A	.	Y
67	near-2n	1	1	A	P	D	.	Y
68	near-2n	1	1	NA	P	A	.	Y

69	near-4n	1	0	NA	NP	A	Y	.
70	near-4n	0	1	A	P	D	Y	Y
71	near-4n	1	1	NA	NP	A	Y	Y
72	near-4n	0	1	NA	P	A	Y	.
73	near-4n	1	0	A	P	D	.	Y
74	near-4n	1	1	A	P	D	.	Y

MYCN amplification status: **NA **= not amplified, **A **= amplified. Disease status: **NP **= no disease progression, **P **= disease progression. Survival status: **A **= alive, **D **= dead. Microarray and validation analyses: Y = cases analyzed.

Twenty-one samples (9 stages 1, 2, and 3; 1 stage 4s; and 11 stage 4) of the original MSKCC NBT cohort included in the gene profiling analysis and an independent set of 25 primary NBT specimens (12 stage 1, 2, and 3, 2 stage 4s, and 11 stage 4) obtained at diagnosis from 3 Spanish institutions (HSJD, Barcelona; Hospital La Paz, Madrid; and Department of Pathology, University of Valencia) were available for validation analyses (Table 1). Normal control DNA was obtained from the National DNA Bank of Spain.

All tumour-specimens were evaluated by the same pathologists (WG and NP) to assess tumour cell content, only tumours with > 70% were included in the study.

### DNA content analysis

The modal DNA content was determined by flow cytometry DNA analysis on nuclei isolated from paraffin sections using the method of Hedley modified [8]. DNA index (DI) was expressed as the ratio of tumour DNA content/standard DNA fluorescence; near-diploid DI = 0.90–1.20; near-triploid DI = 1.21–1.75; near-tetraploid DI = 1.76–2.20.

### Gene expression profiling

Gene expression profiling was performed of 49 primary NBT samples (22 near-triploid, 23 near-diploid and 4 near-tetraploid) using Affymetrix GeneChip Human Genome U95 Set™ Arrays, as previously reported [9]. Microarray data and sample annotations have been deposited in the caArray database .

### Differential gene expression analysis

Genes with high variability within samples were selected by pair-wise comparison analyses performed by adjusting the type-I error for multiple tests (Step-down permutation (SDP) [10], and False Discovery Rate (FDR) [11]), and with no type-I error adjustment (Raw method). The cut-off Family-wise error applied to select significant genes by means of the T-test for independent data, a univariate screening supervised procedure, was equivalent for all three methods: < 0.1, < 0.05 and < 0.01. Hierarchical clustering analyses were performed for the differentially expressed genes for all the methods of adjustment of Type-I error and cut-off of P-values, using a multivariate unsupervised method, taking into account the relationship between gene expressions. Fisher's exact test and 95% bilateral confidence interval using Wilson method were used to evaluate the proportion with which chromosomes were represented in the selected gene sets in comparison to chromosome representation within the Affymetrix GeneChip U95Av2. Statistical analyses were performed using SAS 9.1 and JMP 5.1 (SAS Institute Inc) for Windows and CIA 2.1.1.

### Gene Ontology annotation categories

Gene Ontology (GO) annotation categories were analyzed using *explore *GeneOntology (*e*GOn v2.0) in *Gene Tools *web service  to create a biological profile of the differentially expressed genes. Overrepresented GO terms were determined statistically by Fisher's exact test (*P *< 0.01) and adjusted FDR < 0.01.

### Quantitative Real-time PCR (Q-PCR)

Quantification of transcript levels using Q-PCR was performed of 13 genes located on chromosomes 1 and 17 (see Additional file 1). Concomitant quantification of gene copy number was performed for a set of these genes (see Additional file 1). MYCN gene copy number was analyzed by Q-PCR, and FISH when needed. Validation analyses were performed on 46 primary NBT specimens (see patients and samples).

Q-PCR reactions and quantification, using the ΔΔC_T _relative quantification method, were performed on an ABI Prism 7000 Sequence Detection System using TaqMan^® ^Assay-on-Demand Gene Expression products, according to the manufacturer's protocols (Applied Biosystems, US). All experiments included no template controls and were performed in duplicate and repeated twice independently. Transcript levels were measured relative to 3 normal tissue samples (adrenal gland, lymph node and bone marrow) and normalized to TATA box binding protein (TBP), hypoxantine phosphoribosyltransferase 1 (HPRT1) and succinate dehydrogenase complex, subunit A (SDHA) expression values. Endogenous control genes were chosen on the basis of recent publications regarding accurate normalization of real-time quantitative RT-PCR in primary neuroblastoma [12,13]. These genes are reported within the most stable set of endogenous control genes. Gene copy number quantification was performed as reported previously [14]. Gene copy number was calculated relative to placental DNA using the B-Cell maturation factor (BCMA) as reference gene. The validity of BCMA as reference gene in our cohort of NBTs was determined by copy number ratio: BCMA _NB tumour test sample_/BCMA _placenta calibrator sample_. The ratio measured was equal to 1.0016; (tumour DNA 1.0012 ± 0.13 SD)/(placental DNA 0.9996 ± 0.05).

### Fluorescent *in situ *hybridization (FISH)

FISH was assayed on 4 μm sections of Tissue-Micro-Array (TMA) of formalin-fixed paraffin-embedded NBT samples corresponding to the validation set, and partially matching the MSKCC series described above. Tissue microarrays included only tumour areas showing > 90% of tumour cells. Sections were washed with 2× SSC buffer and fixed in 4% paraformaldehyde in PBS. DNA-probes, CEP 17 Alpha (Ref: 32-112017;Vysis, IL, USA) LSI p53 (Ref:30-190008;Vysis) and/or LSI 1p36 (Ref:30-231004;Vysis), were denatured at 73°C, 5 min., applied to tissue sections and simultaneously denatured using the Hybridizer (DAKO) at 90°C, 4 min. Hybridization was performed for 16 h at 37°C in a humid chamber. Slides were then washed with Buffer post-hybridization (Master Diagnostica, Granada, Spain) and stained with DAPI (6-diamidino-2-phenylindole) and mounted with Vectashield H-1000 medium (Vector). One hundred nuclei were evaluated for each core. Results were recorded as percentage of nuclei present in the sample having each probe signal pattern. Cell populations < 5% of abnormal cells were not scored as significant. Microscope Magnification ×1000.

### Array comparative genomic hybridization (aCGH)

Whole genome BAC-aCGH studies were performed using the Sanger 1 Mb clone set (kindly provided by Dr. K. Szuhai LUMC, The Netherlands). BAC/PAC clones were added to increase resolution for regions of interest: full genomic coverage clones for chromosome 17 (CHORI) and chromosome 11 (BAC/PAC isolated DNA, kindly provided by Dr. J. San Miguel, CIC, Salamanca), and 19q13 enriched medium-coverage set (Invitrogen, CA, USA and kindly provided by Dr. JC Cigudosa, CNIO, Spain). BAC DNA was extracted, amplified by DOP and Aminolinking-PCR and spotted in triplicate onto Codelink slides (Amersham Biosciences, GE, USA).

Tumour and reference DNA (an equimolar DNA pool from 40 healthy donors, obtained from the Spanish National DNA Bank) was Cy5/Cy3-dCTP (Amersham, GE) labelled using a non-commercial Random Priming kit composed by Random Octamers dissolved in Eppicentre Exo-Minus Klenow buffer, a dNTPs mix depleted in dCTP and Exo-Minus Klenow enzyme (Eppiocentre). Labelled DNA was purified through Illustra G-50 Microspin Columns, mixed and then precipitated along with Cot DNA (Roche). Hybridization was performed for 48 hours at 42°C and probe excess removed.

#### Imaging acquisition and data analysis

Log_2 _data was acquired using Axon 4000B scanner and GenePix software. Normalization was done with GenePix software using the mean of the median of ratios of all the autosomal features in the array, excluding those removed by the quality flagging scripts. Gpr files were subsequently processed with Bioconductor packages (CRAN) incorporating scripts for removing SD > 0.2 and GenePix flagged spots. DNA copy algorithm and Merge Levels scripts (both implemented in snap CGH package) were applied for segmentation of the data. A graded colour code adjusted to the log_2 _rank of each individual plot was assigned to define the segments found by the applied algorithm. Universal threshold cut-off values for defining gain/loss were not applied because of subpopulation clonal heterogeneity, ploidy, and percentage of neuroblastic cells, which varied from one sample to another. Due to this, plots were evaluated independently by visual examination and results were depicted using a graded colour code adjusted to the log_2_ rank of each plot, assigning a colour grade to every segment found by the segmentation algorithm.

## Results

### Differential gene expression analysis

**Figure 1:**
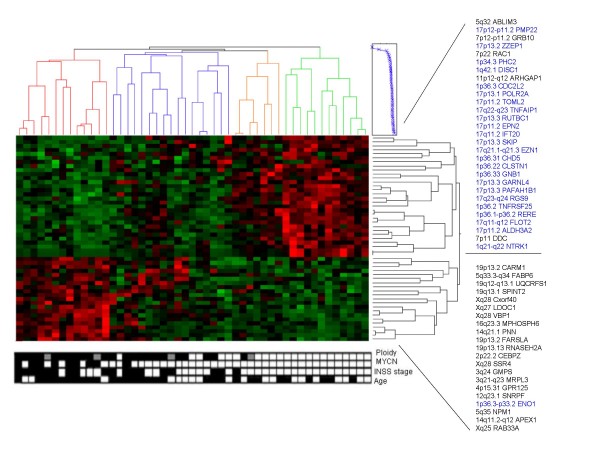
**A heatmap illustrating the distinct expression profiles of 49 NB primary tumours with varying ploidy status**. Gene expression profiles visualized according to 51 differentially expressed genes [FDR < 0.05]. **(Right) **Gene dendrogram is divided in 2 main gene clusters. Top cluster: genes displaying higher expression in near-triploid tumours; a statistically significant proportion of genes map to chromosome 1 (p = 0.01) and chromosome 17 (p < 0.0001) (Blue). Bottom cluster: genes with higher expression in near-diploid/tetraploid NBTs. **(Bottom) **Filled in boxes: **Ploidy: **black = near-diploid, empty white boxes = near-triploid, grey = near-tetraploid NBTs; **MYCN: **black = amplified, white = not amplified; **Age: **black > 12 months, white < 12 months; **INSS: **black = Stage 4 NBTs, white = stages 1, 2, 3, and 4S.

Gene expression analysis was performed on a spectrum of 49 NBTs with varying DNA content (22 near-triploid, 23 near-diploid and 4 near-tetraploid). Owing to reduced number of near-tetraploid cases included in this study and taking into account the reported biological and clinical similarities with near-diploid NBTs [15,16], near-diploid and near-tetraploid NBTs were combined in one group. Pair-wise comparison analyses of near-triploid (n = 22) *versus *near-diploid/tetraploid (n = 27) NBTs revealed small sets of differentially expressed genes when using a stringent correction for multiple sampling, (6 genes [FDR < 0.01] and 12 genes [SDP < 0.1]) (see Additional file 2). Interestingly, all genes showing a higher expression in the near-triploid group mapped to chromosome 17 (see Additional file 2). Less stringent multiple testing corrections selected a larger set of differentially expressed genes, (51 genes [FDR < 0.05] (Fig. 1) and 254 genes [FDR < 0.1] (see Additional file 2). Again, this resulted in a statistically significant proportion of genes mapping to chromosomes with described recurrent abnormalities in NBTs; chromosome 1 (p = 0.01) and chromosome 17 (p < 0.0001) (Fig. 1). Chromosomal region specificity was observed since the majority of chromosome 1 and 17 differentially expressed genes spread over 1p36-p22.1 and 17p13-17q21 (Fig. 1; see Additional file 2). The majority showed higher expression in near-triploid NBTs; 92% (CI: 78% to 97%) of chromosome 1 genes and 91% (CI: 76% to 96%) of chromosome 17 (see Additional file 2). Only 8% (CI: 2% to 21%) probe sets for genes located on chromosome 1, ENO1 (1p36.2), CCT3 (1q23) and C1orf107 (1q32.2), and 9% (CI: 3% to 23%) for genes on chromosome 17, MAC30 (17q11.2) and NME1 (17q21.3), showed a higher expression within near-diploid/tetraploid NBTs.

The Gene Ontology biological profile of genes with higher expression in near-diploid/tetraploid NBTs showed enrichment for genes related to protein, macromolecular and nucleic acid biosynthesis, such as, NME1, ATP5I, ATP5C1, NME4, TYMS and GMPS. Whereas, near-triploid tumours included genes involved in vesicle mediated transport, cell communication, signal transduction, nervous system development and regulation of small GTPase mediated signal transduction. A large portion of these genes mapped to chromosomes 1 and 17 (60–100%), among these RERE, CHD5, CLCN6, CDC42BPA, NTRK1, ARHGEF11, PMP22, VAMP2, GARNL4, MAP2K4 and FLOT2.

### Quantitative Real-time Polymerase Chain Reaction (Q-PCR)

**Figure 2:**
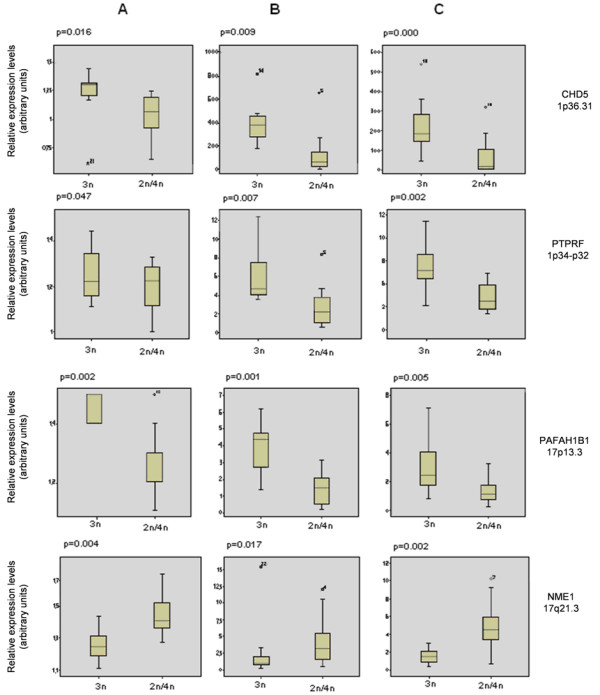
**Quantitative real-time PCR validation of microarray gene expression data**. Comparison of gene expression levels of 5 representative genes located on chromosomes 1 and 17. **A**. Microarray gene expression data in 49 NBT from MSKCC. Gene expression data were log-transformed and normalized to TBP expression levels; **B**. Q-PCR gene transcript quantification in 21 NBTs from MSKCC; **C**. Q-PCR gene transcript quantification in 25 NBTs from Spanish institutions. Results were compared by two-tailed independent-sample *t *test using SPSS v.14.0 for Windows (SPSS, Chicago, IL). Expression data are shown as box plots (SPSS v.14.0).

Quantification of transcript levels of 13 differentially expressed genes, located mainly on the chromosomal regions 1p36 and 17p13-q21, was performed on two separate groups of NBT specimens: 21 primary NBTs from the original MSKCC cohort as well as on an independent set of 25 NBTs (Table 1). Expression levels identified by Q-PCR confirmed the microarray data in both sets of NBTs (Fig. 2A, B and 2C).

Four genes located on chromosomes 1 and 17 were further analyzed for gene copy number by DNA Q-PCR analysis in 27 cases (Tables 2 and 3; see Additional file 3). Near-triploid NBTs (n = 13) showed, both for chromosome 1 and 17, fold values consistently higher (≥ 1.3-fold) than normal reference gene values, and were considered to represent a minimum trisomic gene copy number. Only case # 2 (Table 2; see Additional file 3) showed 0.8–1.1-fold values reflecting a possible loss of 1p36, subsequently confirmed by FISH and aCGH results. Near-diploid/tetraploid NBTs (n = 13) displayed a wider range of values (0.5–2.7-fold), indicative of losses and gains within a more heterogeneous clonal population, as shown by FISH results. Tumour clonal heterogeneity may often confound analyses performed on the bulk of the tumour specimen and could explain some discrepancies between ploidy and gene copy number.

**Table 2:** Results of FISH, aCGH, Q-PCR analyses of chromosome 1, displayed in relation to NBTs ploidy status

**Case Number**	**Ploidy**	**MYCN**	**FISH Chromosome 1**	**a CGH Chr. 1**			**Q-PCR Gene copy No. (fold change)**		**Disease Status**	**Survival Status**
			Cell % (#DNA probe signals: LSI 1p36: LSI 1q25)	p	cen	q	GNB1 (1p36.33)	RERE (1p36.1)		

1	near-3n	NA	n.e	G	G	G	1.6	3.2	NP	A
2	near-3n	NA	50 (2:2), 20 (3:3), 15 (1:3), 15 (2:3)	L	-	-	0.8	1.1	NP	A
3	near-3n	NA	60 (2:2), 40 (3:3)	-	-	-	1.5	2	NP	A
4	near-3n	NA	5 (2:2), 95 (3:3)	G	G	G	2.6	2.4	NP	A
5	near-3n	NA	40 (2:2), 60 (3:3)	-	-	-	1.4	1.3	NP	A
6	near-3n	NA	50 (2:2), 50 (3:3)	G	G	G	1.4	3	NP	A

7	near-2n	A	n.e.	L	-	-	0.5	2.2	P	D
8	near-2n	NA	n.e.	G	G	G	1.6	1.5	NP	A
9	near-2n	NA	95 (2:2), 5 (3:3)	n.e	n.e	n.e	0.7	0.5	NP	A
10	near-2n	NA	100 (2:2)	-	-	-	0.7	0.5	P	D
11	near-2n	NA	35 (2:2), 65 (1:3)	n.e	n.e	n.e	1	2.3	P	D

12	near-4n	A	51 (1:2), 30 (2:2), 19 (1:3)	-	-	G	0.5	0.6	P	D
13	near-4n	A	60 (2:2), 30 (3:3), 10 (4:4)	-	-	-	1.3	2.7	P	D

Thirteen representative cases drawn from the HSJD cohort analyzed by FISH, aCGH and Q-PCR of chromosome 1. **n.e **= not evaluable results. MYCN amplification status: **NA **= not amplified, **A **= amplified. Disease status: **NP **= no disease progression, **P **= disease progression. Survival status: **A **= alive, **D **= dead. FISH: results are displayed as percentage of cells exhibiting the observed number of DNA probe signals, and exact number of signals for the DNA probes used: chromosome 1 (LSI 1p36 and LSI 1q25 DNA probes) and chromosome 17(LSI 17p13.1 and CEP 17 DNA probes). Array CGH: **p **and **q **= chromosome arms, **cen**. = centromeric; **G **= chromosome gain, **L **= chromosome loss. Q-PCR: gene copy number fold changes are determined by the ΔΔC_T _relative quantification method.

**Table 3:** Results of FISH, aCGH, Q-PCR analyses of chromosome 17, displayed in relation to NBTs ploidy status

**Case Number**	**Ploidy**	**MYCN**	**FISH Chromosome 17**	**a CGH Chr. 17**			**Q-PCR Gene copy No. (fold change)**		**Disease Status**	**Survival Status**
			Cell % (# DNA probe signals: LSI 17p13.1: CEP 17)	p	cen	q	RUTBC1 (17p13.3)	NME1 (17q21)		

1	near-3n	NA	n.e	G	G	G	2.3	2.5	NP	A
2	near-3n	NA	45 (2:2), 55 (3:3)	G	G	G	1.5	1.5	NP	A
3	near-3n	NA	45 (2:2), 55 (3:3)	G	G	G	1.3	1.4	NP	A
4	near-3n	NA	30 (2:2), 70 (3:3)	G	G	G	3.6	2.2	NP	A
5	near-3n	NA	50 (2:2), 50 (3:3)	G	G	G	1.6	1.3	NP	A
6	near-3n	NA	50 (2:2), 50 (3:3)	G	G	G	1.4	1.5	NP	A

7	near-2n	NA	5 (1:1), 80 (2:2), 10 (3:3), 5 (4:4)	n.e	n.e	n.e	0.7	1.4	NP	A
8	near-2n	NA	33 (1:1), 66 (2:2)	-	-	-	0.8	0.7	P	D
9	near-2n	A	7 (1:1), 7 (2:1), 60 (2:2), 20 (1:2), 6 (2:3)	-	-	G	1.1	1.4	P	D
10	near-2n	NA	80 (2:2), 15 (2:3), 5 (3:3)	-	-	G	1.2	1.5	NP	A
11	near-2n	NA	28 (1:1); 11 (2:1), 56 (2:2), 5 (3:2)	n.e	n.e	n.e	1.1	0.9	P	D

12	near-4n	A	45 (2:2); 55 (3:3)	-	-	G	1	1.4	P	D
13	near-4n	A	45 (2:2), 45 (3:3), 10 (4:4)	G	G	G	2.2	1.9	P	D

Thirteen representative cases drawn from the HSJD cohort analyzed by FISH, aCGH and Q-PCR of chromosome 17. **n.e **= not evaluable results. MYCN amplification status: **NA **= not amplified, **A **= amplified. Disease status: **NP **= no disease progression, **P **= disease progression. Survival status: **A **= alive, **D **= dead. FISH: results are displayed as percentage of cells exhibiting the observed number of DNA probe signals, and exact number of signals for the DNA probes used: chromosome 1 (LSI 1p36 and LSI 1q25 DNA probes) and chromosome 17(LSI 17p13.1 and CEP 17 DNA probes). Array CGH: **p **and **q **= chromosome arms, **cen**. = centromeric; **G **= chromosome gain, **L **= chromosome loss. Q-PCR: gene copy number fold changes are determined by the ΔΔC_T _relative quantification method.

**Figure 3:**
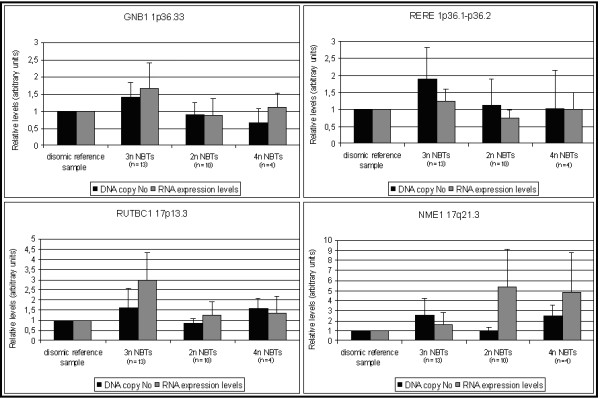
**Comparison between DNA copy number and gene expression levels analyses**. Gene expression levels and gene copy number are exhibited as mean values in accordance with NBT ploidy subgroups. Correlation between DNA gene copy number and expression levels was observed in those analyzed genes that displayed in the microarray analysis higher expression levels in near-triploid NBTs.

Comparison between DNA gene copy number and expression levels (Fig. 3) revealed an overall linear correlation for those analyzed genes that displayed in the microarray analysis higher expression levels in near-triploid NBTs. Conversely, NME1 gene, as from microarray results, showed low expression values, closer to the disomic reference sample expression, in near-triploid NBTs, and high fold increase in mRNA levels in near-diploid and tetraploid cases.

### Fluorescent *in situ *hybridization (FISH)

Interphase FISH using the DNA probes LSI 1p36 and LSI 1q25 was performed on 13 primary NBTs drawn from the HSJD cohort; four cases were not evaluable (Table 2). According to chromosome 1 status, near-triploid and near-diploid/tetraploid NBTs were characterized by intratumoural heterogeneous cell population content. Only 1 case showed uniform distribution of probe signals within cells of the tumour specimen (case #10, Table 2). All but one of the near-triploid NBTs were constituted of clonal populations with two LSI 1p36 and LSI 1q25 signals (2:2) and/or three (3:3) DNA probe signal, ranging from 40–60% and 40–100% of the cells, respectively. Case # 2 was the only near-triploid NBT that exhibited a chromosome 1p36 loss in 30% of cells, confirmed by aCGH and Q-PCR. Even higher intratumoural heterogeneity was observed in near-diploid/tetraploid NBTs.

Chromosome 17 FISH using centromeric CEP 17 and LSI p53 (17p13.1) DNA probes, was performed on 53 primary NBTs (13 cases from the HSJD cohort, Table 3, and 40 cases from MSKCC, Table 4). Based on chromosome 17 status, near-triploid tumours were constituted of two (2 CEP 17 and 2 LSI p53 signals, 2:2), three (3:3) and four (4:4) chromosome 17 signals clonal populations that ranged from 10–55%, 24–70% and 7–45% of the cells, respectively. Near-diploid/tetraploid NBTs were composed by a more heterogeneous cell population, with a high incidence of chromosomal structural abnormalities. In a large portion of these tumours, alongside with the two (2:2) DNA probe signal clonal populations (6%–100% of cells), the aneuploid cell population counterpart constituted a significant and heterogeneous portion of cell population (Tables 3 and 4).

**Table 4:** Chromosome 17 Fluorescence *in situ *Hybridization results of 40 NBTs obtained from MSKCC, displayed in relation to NBTs ploidy status

**Case Number**	**Ploidy**	**MYCN**	**FISH Chromosome 17**	**Disease Status**	**Survival Status**
Cell % (# DNA probe signals: LSI 17p13.1: CEP 17)					

1	near-3n	NA	23 (2:2), 44 (3:3), 33 (4:4)	NP	A
2	near-3n	NA	50 (2:2), 50 (3:3)	NP	A
3	near-3n	NA	16 (2:2), 41 (3:3), 43 (4:4)	NP	A
4	near-3n	NA	34 (2:2), 42 (3:3), 24 (4:4)	NP	A
5	near-3n	NA	33 (2:2), 50 (3:3), 17 (4:4)	P	A
6	near-3n	NA	25 (2:2), 60 (3:3), 15 (4:4)	NP	A
7	near-3n	NA	31 (2:2), 46 (3:3), 23 (4:4)	NP	A
8	near-3n	NA	35 (2:2), 52 (3:3), 13 (4:4)	NP	A
9	near-3n	NA	23 (2:2), 54 (3:3), 23 (4:4)	NP	A
10	near-3n	NA	13 (3:3), 66 (3:4), 21 (5:5)	NP	A
11	near-3n	NA	16 (2:2), 48 (3:3), 36 (4:4)	P	A
12	near-3n	NA	35 (2:2), 58 (3:3), 7 (4:4)	NP	A
13	near-3n	NA	46 (2:2), 24 (3:3), 30 (4:4)	NP	A
14	near-3n	NA	22 (3:3), 62 (4:4), 16 (4:5)	P	D
15	near-3n	NA	10 (2:2), 29 (3:3), 45 (4:4), 16 (5:5)	P	D

16	near-2n	NA	5 (1:1), 65 (2:2), 5 (1:2), 10 (3:3), 5 (2:3), 5 (4:4), 5 (3:4)	P	D
17	near-2n	NA	100 (2:2)	P	D
18	near-2n	NA	95 (2:2), 5 (3:3)	P	D
19	near-2n	NA	31 (CEP 2), 50 (CEP 3), 18 (CEP 4)	NP	A
20	near-2n	NA	25 (1:1), 75 (2:2)	P	D
21	near-2n	NA	100 (2:2)	P	D
22	near-2n	NA	n.e	P	D
23	near-2n	NA	10 (CEP 1), 40 (CEP 2), 38 (CEP 3), 12 (CEP 4)	P	A
24	near-2n	NA	80 (2:2), 10 (1:2), 5 (3:3), 5 (2:2)	P	A
25	near-2n	NA	100 (2:2)	P	D
26	near-2n	NA	25 (1:1), 75 (2:2)	P	D
27	near-2n	NA	100 (2:2)	P	D
28	near-2n	NA	5 (2:1), 74 (2:2), 5 (1:2), 5 (3:3), 6 (2:3), 5 (4:4)	P	D
29	near-2n	NA	10 (2:1), 70 (2:2), 15 (1:2), 5 (3:3)	P	D
30	near-2n	NA	n.e	P	D
31	near-2n	A	20 (2:2), 32 (3:3), 12 (4:3), 20 (4:4), 16 (3:4)	NP	A
32	near-2n	A	40 (1:1), 60 (2:2)	NP	A
33	near-2n	A	n.e	NP	A
34	near-2n	A	100 (2:2)	P	A
35	near-2n	A	35 (2:2); 5 (3:2), 20 (3:3), 5 (2:3), 30 (4:4), 5 (3:4)	P	D
36	near-2n	A	60 (2:2); 5 (3:2), 25 (3:3), 5 (4:3), 5 (4:4)	P	D
37	near-2n	A	10 (1:1), 90 (2:2)	P	D

38	near-4n	NA	29 (2:2), 6 (3:3), 8 (4:3), 37 (4:4), 20 (3:4)	NP	A
39	near-4n	NA	49 (2:2), 37 (3:3), 9 (2:3), 5 (3:4)	NP	A
40	near-4n	NA	6 (2:2), 20 (3:3), 5 (4:3), 46 (4:4), 18 (5:5), 5 (4:5)	P	A

**n.e **= not evaluable results. MYCN amplification status: **NA **= not amplified, **A **= amplified. Disease status: **NP **= no disease progression, **P **= disease progression. Survival status: **A **= alive, **D **= dead. FISH: results are displayed as percentage of cells exhibiting the observed number of DNA probe signals, and exact number of signals for the DNA probes used: chromosome 1 (LSI 1p36 and LSI 1q25 DNA probes) and chromosome 17(LSI 17p13.1 and CEP 17 DNA probes). Array CGH: **p **and **q **= chromosome arms, **cen**. = centromeric; **G **= chromosome gain, **L **= chromosome loss. Q-PCR: gene copy number fold changes are determined by the ΔΔC_T _relative quantification method.

**Figure 4:**
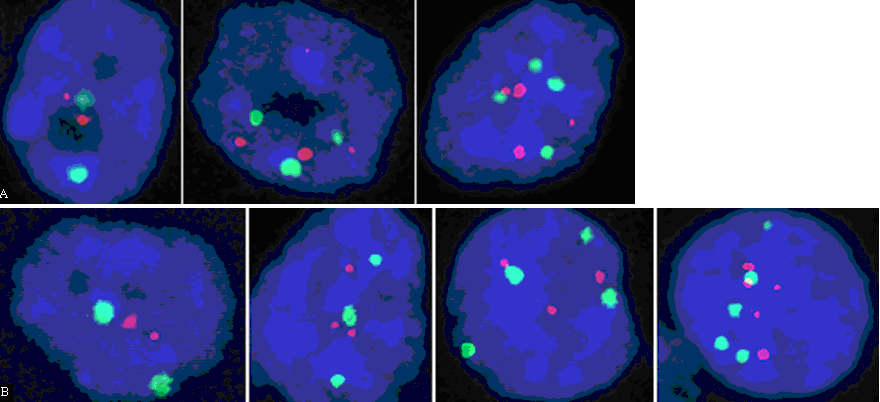
**FISH analysis. Intra-tumoural cell heterogeneity, cancer cells exhibit different alterations of chromosome 17**. FISH analysis using probes for chromosome 17 (red, LSI p53; green, CEP 17) showing different cellular populations within the same NBT in terms of probe signal numbers. In the panels are reported two representative NBT cases; **A**. near-triploid NBT; **B**. near-diploid case. Five signal cells in this sample were very rare populations (< 5%) and are not displayed in Table 3.

Intratumoural clonal heterogeneity was observed in all the FISH analyses (Fig. 4).

### Array comparative genomic hybridization (aCGH)

**Figure 5:**
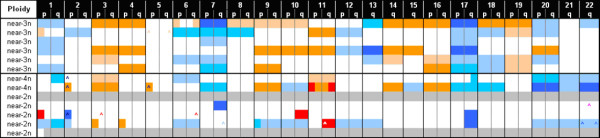
**Array-Comparative Genomic Hybridization (aCGH) results of 13 NBTs obtained from HSJD**. Results are displayed according to tumour ploidy status. Chromosome alterations are visualized as a graded colour code adjusted to the log_2 _rank of each individual plot assigned to define chromosomal segment alterations. Filled boxes: from orange to pink colour shades represent increasing chromosomal copy number gains, whereas, from light blue to dark blue colour shades indicate chromosome losses. White colour boxes represent no detected chromosome change. Grey colour boxes represent not evaluable results.

Genome array CGH was performed for 13 cases, drawn from the HSJD validation set of NBTs, with complete FISH and Q-PCR analyses (Tables 2 and 3; Fig. 5). Near-triploid NBTs exhibited the highest incidence of specific chromosomal alterations, with consistent gain or loss of whole chromosomes, being chromosomes 7 and 17 the most frequently gained (83% and 100% cases, respectively), whilst, chromosomes 3, 4, 9, 14, 16 (50% cases), and 19 (67% NBTs) were among the most frequently lost, although the set of cases is not large enough for statistically significant results. Chromosome 1p loss was observed only in one case (case# 2, Table 2), a near-triploid stage 4s tumour.

Specific near-diploid/tetraploid copy number alterations were characterized by a more heterogeneous pattern of chromosomal aberrations than those of near-triploid, being partial chromosomal segment alterations much more frequent than in near-triploid tumours (Fig 5; see Additional file 4). Partial loss of 11q and partial gain of 17q were only observed in near-diploid/tetraploid samples and never in near-triploid NBTs. Chromosome 20 showed a common pattern being one of the most frequent gains both in near-diploid and near-triploid NBTs. MYCN amplification was absent in near-triploid cases and shared by near-diploid/tetraploid cases.

Further copy-number alterations that did not reach the maximum log_2 _values, but were clearly distinguishable in terms of segmentation algorithm, were detected in the array CGH plots and could reflect higher intratumoural clonal heterogeneity (data not shown).

## Discussion

Aneuploidy is ubiquitous in cancer and has been linked to cell proliferation, cell differentiation and prognosis. The karyotypes of most tumours are aneuploid, meaning that chromosomes, which carry thousands of genes, are structurally rearranged, duplicated, broken or entirely missing.

Gain of chromosome 17 is one of the most frequent genetic abnormalities observed in NBTs, and may involve either the entire chromosome or partial gain of the distal segment 17q21-qter [17]. Unbalanced translocations, characteristic of near diploid NBTs or tumours with structural rather than numerical chromosome aberrations, are thought to arise from DNA double strand breaks repaired erroneously, suggesting an impaired DNA maintenance or repair pathway [18]. On the other hand, abnormalities in the mitotic segregation of chromosomes are thought to underlie the numerical aberrations characteristic of near-triploid, good prognostic, NBTs. Both mechanisms define the type of aneuploidy behind each of the subgroups of NBTs, determining the kind of genetic aberrations as well as the biological behaviour of each NBT subtype.

Gene expression profiling of NBTs with different ploidy status, near-triploid or near-diploid/tetraploid, enabled us to identify distinct expression profiles associated with each subgroup. Interestingly, a statistically significant proportion of genes shown to be differentially expressed mapped to chromosomes described to be recurrently altered in NBTs, chromosomes 1 and 17 [17]. Chromosomal region specificity was also observed for these differentially expressed genes since the majority spread predominantly over the chromosomal regions 1p36-p22.1 and 17p13-17q21. Besides, over 90% of these genes displayed higher expression levels in near-triploid tumours. Only two genes mapping to chromosome 17, MAC30 and NME1, exhibited a higher expression level in near-diploid/tetraploid NBTs. MAC30 gene encodes for a meningioma-associated protein, highly expressed in several types of tumours, but, with unknown clinicopathological and biological significance. The product of the NME1 gene, the nm23A protein, is a nucleoside diphosphate kinase, whose expression has been related to cell proliferative activity [19]. Whereas reduced expression of NME1 is associated with a high potential for metastasis in some tumour types, like breast cancer and melanoma, its expression is increased in aggressive NBTs [20].

Genome array CGH, together with FISH and Q-PCR results, confirmed the association of specific chromosomal abnormalities with each of the NBTs subgroups. Therefore, it is not unreasonable to assume that these specific chromosomal alterations are associated with the observed gene expression profiles. The highly significant and strikingly persistent chromosomal localization of the differentially expressed genes made us hypothesize about which transcriptional regulation mechanisms can underlie these gene expression patterns. As a result of aneuploidy, cells possibly produce imbalanced expression of large sets of genes that are amplified or lost. Such gross imbalances would inevitably disrupt critical cellular circuits and destabilize regulatory pathways and cellular structures. It has been assumed that gene dosage effects may play a role in the pathogenesis of malignant diseases. Variations of the transcriptome due to alterations of the gene dosage have been described *in vitro *[21], *in vivo *[22] and in human pathologies such as trisomies 13 and 21 [23]. In our hands, when comparing gene expression levels with gene copy number of a set of differentially expressed genes located at chromosomes 1p36 and 17q13-q21, we observed a concordance between copy number and mean expression values in all those analyzed genes that displayed in the microarray analysis higher expression levels in near-triploid NBTs. In contrast, NME1 gene, as from microarray results, showed low expression values, close to the disomic reference sample expression, in near-triploid NBTs, and high fold increase in mRNA levels in near-diploid/tetraploid cases. NME1 gene has been identified as one of the MYCN targets. Correlation between MYCN overexpression and upregulation of NME1 expression has been reported both in NBTs and neuroblastoma cell lines [24]. In our experience, all MYCN amplified NBTs, displaying MYCN overexpression, as well as near-diploid cases with increased copy number of chromosome 17q, showed high NME1 expression levels. However, NME1 overexpression was also observed in 2 near-diploid MYCN single copy cases, with low MYCN expression and no 17q gain. This suggests that in NBTs NME1 gene expression is only partly dependent on gene copy number and MYCN expression, and therefore implies the existence of other mechanisms of NME1 transcriptional regulation.

Recently, we reported that clonal ploidy heterogeneity is present in virtually every single loco-regional, near-triploid NBT, and detected the existence of clonal DNA content heterogeneity and evolution [25,26]. In this report our results underscore the clonal heterogeneity of all NBTs, with a marked complexity in the near-diploid/tetraploid tumours. Furthermore, clonal variations reflected in the array CGH plots as copy-number alterations with varying log_2 _values, could unveil the presence of subpopulations emerged during tumour development. These cellular subpopulations are likely to be the cause of the high cell heterogeneity also observed in the FISH analyses. These findings are important in emphasizing the cellular heterogeneity and karyotypic complexity (aneuploidy) generally associated with malignant tumours, but need a more detailed understanding of their significance.

## Conclusion

We have found that NBTs with different cellular DNA content display specific transcriptional profiles suggesting that near-diploid/tetraploid and near-triploid NBTs result from two different mechanisms of aneuploidy driving tumourigenesis. A large number of the differentially expressed genes participate in cell differentiation pathways and map to specific chromosomal regions recurrently involved in unbalanced translocations, gains and losses in NBTs. Our results demonstrate that these specific genetic abnormalities are complex, heterogeneous, and translate into a gene expression profile that defines the biological behaviour of each type of NBT.

## Abbreviations

NBTs: neuroblastic tumours; MIBG: Meta-iodobenzylguanidine; LOH: loss of heterozygosity; MSKCC: Memorial Sloan-Kettering Cancer Center, New York; HSJD: Hospital Sant Joan de Déu, Barcelona; Children's Oncology Group: COG; CT: computed tomography; INSS: International Neuroblastoma Staging System; INPC: International NB pathology committee; CNS: central nervous system; Q-PCR: Quantitative real-time polymerase chain reaction; aCGH: array-Comparative Genomic Hybridization; FISH: Fluorescence *in situ *hybridization.

## Competing interests

The authors declare that they have no competing interests.

## Authors' contributions

CL and JM are responsible for the initial conception and overall hypothesis of this study. CL, IG and JM are responsible for the design of this manuscript, including the original draft and subsequent revisions and design of this manuscript. CdT assisted with the initial concept and was involved with the draft and revisions of this manuscript; provided guidance for many of the experiments. NKC and WLG are responsible for the procurement and cryopreservation of NBT tissue specimens derived from MSKCC. ER, IG, SA, HB and JM were responsible for the procurement and cryopreservation of NBT tissue specimens derived from the Spanish institutions. WLG and NP evaluated all tumour specimens for tumour staging classification and to assess tumour content. CL, NKC, WLG, and JM are responsible for patient clinico-biological database management and for microarrays studies. NKC and WLG were involved in the drafting and revision of this manuscript. IG and CL are responsible for the quantitative PCR experiments. CM and EdA are responsible for the FISH and aCGH analyses and were also involved with the interpretation of data, draft and revision of this manuscript. ET performed the flow cytometry DNA analysis. CL, GD, JR and IG performed the statistical analysis and interpretation of the data derived from all the samples. HB and SA assisted with valuable technical assistance for experiments associated with this manuscript. All were also involved in the drafting and revisions for this manuscript. All authors read and approved the final manuscript.

## Pre-publication history

The pre-publication history for this paper can be accessed here:



## Supplementary Material

Additional file 1 Quantitative Real-time Polymerase Chain Reaction Analysis. List of genes analyzed to determine expression levels and DNA copy number of genes located on chromosomes 1 and 17. Click here for file

Additional file 2 Gene expression profiling of NBTs with different ploidy status. List of differentially expressed genes identified applying different multiple testing corrections. Differentially expressed genes are displayed according to tumour ploidy and chromosomal location. **A**. List of 6 differentially expressed genes [FDR P < 0,01]; B. List of 12 differentially expressed genes [SDP P < 0,1]; C. List of 51 differentially expressed genes [FDR P < 0,05];.D. List of 254 differentially expressed genes [FDR P < 0,1]. Click here for file

Additional file 3 Quantitative Real-time Polymerase Chain Reaction gene copy number analysis and array CGH analysis results. **n.e **= not evaluable results; **n.d**. = not done. MYCN amplification status: **NA **= not amplified, **A **= amplified. Disease status: **NP **= no disease progression, **P **= disease progression. Survival status: **A **= alive, **D **= dead. **Q-PCR**: gene copy number fold changes are determined by the ΔΔC_T _relative quantification method. Array CGH: **p **and **q **= chromosome arms, **cen**. = centromeric; **G **= chromosome gain, **L **= chromosome loss; **- **= no alteration observed. Click here for file

Additional file 4 Array CGH images of NBT with different DNA content. **A**. Near-triploid NBT; **B**. Near-diploid tumour and **C**. Near-tetraploid NBT. Click here for file
