# Supplementary figures and images for: Differential expression of genes mapping to recurrently abnormal chromosomal regions characterize neuroblastic tumours with distinct ploidy status
Source: BMC Med Genomics. 2008 Aug 13;1:36. doi: 10.1186/1755-8794-1-36 (PMC2531130; doi:10.1186/1755-8794-1-36)

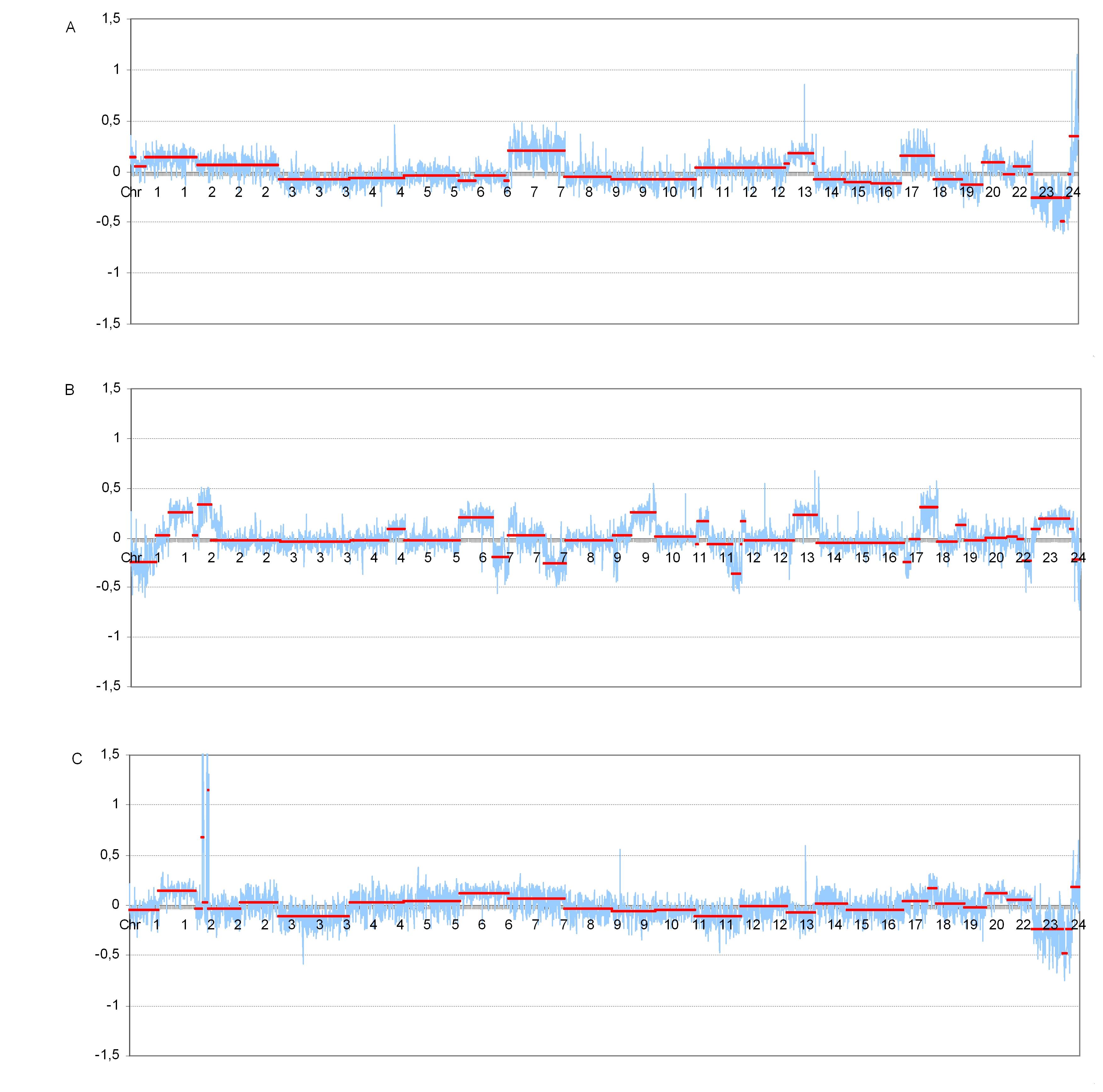

Supplement: Additional file 4 — Array CGH images of NBT with different DNA content. A. Near-triploid NBT; B. Near-diploid tumour and C. Near-tetraploid NBT. [file 1755-8794-1-36-S4.jpeg]
